# Acceleration and Deceleration Profiles: Comparison Between the 5-0-5 Test and Seasonal Peak Player Performance

**DOI:** 10.3390/sports14010009

**Published:** 2026-01-03

**Authors:** Ricardo Pimenta, Hugo Antunes, Fábio Yuzo Nakamura

**Affiliations:** 1 Research Center of the Polytechnic Institute of Maia (N2i), Maia Polytechnic Institute (IPMAIA), Castêlo da Maia, 4475-690 Maia, Portugal; 2 Research Center in Sports Sciences, Health Sciences and Human Development (CIDESD), University of Maia, 4475-690 Maia, Portugal; fnakamura@umaia.pt; 3 Department of Rehabilitation and Optimization of Performance (DROP), Futebol Clube de Famalicão-Futebol SAD, 4760-482 Famalicão, Portugal; hugoduarteantunes@gmail.com; 4 FSI Lab, Football Science Institute, 41, 18016 Granada, Spain; 5 Department of Neurosciences, Biomedicine and Movement Sciences, University of Verona, 37124 Verona, Italy; 6 Graduate Program in Physical Education, Federal University of Pernambuco, Recife 50670-901, Brazil

**Keywords:** change of direction, soccer, locomotor performance, GPS, repeatability

## Abstract

Change-of-direction (COD) capacity is a key performance metric in football due to the high volume of COD actions occurring during match play. This study aimed to (i) evaluate the repeatability of the 5-0-5 test and (ii) examine the relationship between acceleration and deceleration profiles of the 5-0-5 test and in-season peak performance. Nineteen national-level Portuguese football players competing in the under-23 Portuguese National Championship were analysed. Repeatability analysis was conducted using intraclass correlation coefficients (ICC). Paired-samples *t*-tests and Pearson correlations (*r*) were employed to assess within-subject differences and associations between 5-0-5 measures (highest accelerations from a standing start [ACC_S], deceleration prior to the change in direction (DEC_COD), acceleration after decelerating and changing direction [ACC_COD]) and seasonal peak performances (ACC_max_). The in-season peak values were determined from the average of the three maximal values that occurred either in training sessions or in matches. Normalized (relative to seasonal performance) parameters showed good repeatability (ICC = 0.76–0.85). The best ACC_COD and DEC_COD were not significantly different from ACC_max_ [ACC_COD] vs. ACC_max_: (5.04 ± 0.39 m/s^2^ vs. 5.36 ± 0.54 m/s^2^; *p* > 0.05) and DEC_max_ [DEC_COD] vs. DEC_max_: (−6.47 ± 0.26 m/s^2^ vs. −6.35 ± 0.61 m/s^2^; *p* > 0.05), respectively. The average and best 5-0-5 ACC_S performances showed significant, moderate correlations (*r* = 0.48; *p* = 0.040 and *r* = 0.50; *p* = 0.028, respectively) with the players’ peak in-season acceleration performance. Therefore, the best ACC_S and ACC_COD attempts can reflect values above 90% of ACC_max_, while DEC_COD reflects values above 100% of DEC_max_ and may assist in monitoring and tailoring training for this capacity on an individual basis.

## 1. Introduction

Performance testing is an essential procedure across sports disciplines, providing insights into a player’s current fitness relative to specific time points. Change of direction (COD) represents a common physical capacity in football players [1,2,3,4]. Higher acceleration capacity in certain positions has been linked to team success in competitions such as La Liga [5]. Key actions, such as shots on target or recovery runs, involve high-intensity accelerations and have been associated with team success [6]. These findings corroborate the need for individualized training according to the accelerative demands of each position and player characteristics. However, adequate prescription of intensity requires accurate assessment of players’ maximum capacities. Despite the limited ecological validity associated with COD tests [7], they differentiate players of different competitive levels [8]. Typical COD assessment methods involve measuring performance in seconds [8] and commonly using timing gates. Although dual-beam timing gates have been recommended for their greater measurement accuracy and reliability [9], using test time alone as an output limits a comprehensive understanding of COD performance, since COD performance is influenced by multiple factors, such as technique, lower limb strength, acceleration (ACC) capacity, and speed [10].

Partially contributing to a more complex understanding of COD assessment performance, and given that GPS devices are reported to be valid and reliable for measuring high-intensity running metrics [11,12,13], these devices could be used to investigate locomotor parameters during COD tests, such as ACC and deceleration (DEC), obtained during test execution. Although GPS devices are not considered the gold standard for monitoring locomotor performance [14,15], they are commonly used in the daily training and competitive settings in football. Therefore, employing GPS devices during preseason COD assessments could provide coaches with valuable and practical insights into players’ maximal locomotor performance. The application of specific testing sessions to isolate certain physical capacities counterbalances the chaotic nature of football, where unpredictability can mask a player’s true maximal capacity. This was verified, for example, in sprinting capacity, where a maximum sprint value from a 40 m linear sprint test resulted in smaller coefficients of variation (CV: 3.3 ± 2.5 vs. 13.2 ± 6.6%) compared to match settings [16]. Therefore, it is plausible that isolating ACC and DEC performance through testing could be a viable option to obtain reliable and accurate measures of a football player’s maximum accelerative capacities. From a time-efficiency perspective, using some COD tests, such as the 5-0-5 test, could serve as an analytical COD exercise implemented in sessions involving high-volume accelerative maneuvers (e.g., MD-4, MD-3) while also acting as an unobtrusive monitoring tool, enabling continuous tracking of maximal accelerative capacities throughout the season without requiring time-consuming procedures during a microcycle.

To the best of our knowledge, no studies have investigated the repeatability of a COD test [8] when assessed using GPS devices in football, despite the reasonable validity and reliability of GPS technology for quantifying ACC efforts [11,12,13,17]. Moreover, few studies have examined the ACC profiles underlying COD actions or compared these profiles across different time points within a competitive season [18]. Silva et al. (2025) investigated the association between the outcomes obtained from the pro-agility test and the peak accelerations recorded during match play [18]. A strong correlation (r = 0.79) between the pro-agility and 5-0-5 tests has been reported [19], despite inherent differences in their locomotor profiles. However, for the 5-0-5 test, no study has explored the relationship between GPS-derived performance metrics and seasonal ACC dynamics, as done for other performance parameters [20,21,22,23].

The purposes of the present study were to (i) determine the intra-session repeatability of the 5-0-5 test using GPS data and (ii) perform a retrospective comparison between the performance variables obtained during preseason testing and the highest training- or match-derived ACC values recorded throughout the competitive season. We hypothesize that: (i) the 5-0-5 test outcomes will exhibit good-to-excellent intra-session repeatability; and (ii) significant differences will be observed between preseason 5-0-5 testing performance levels and the highest ACC levels achieved during the season.

## 2. Materials and Methods

### 2.1. Participants

A convenience sample of 23 highly trained youth football players (age: 19.08± 1.18 years, weight: 76.23 ± 8.53 kg, height: 1.82 ± 0.08 m) from a team competing in the under-23 Portuguese National Championship (the highest national division for the specific age group, Tier 3 [national level]) [24] was invited to participate in this study. Goalkeepers were excluded from the analysis as their activity profile is very different from the other game positions [25], totalling a sample size of 19 players (age: 19.14 ± 1.24 years, weight: 74.64 ± 7.62 kg, height: 1.81 ± 0.08 m). The study was conducted following the principles of the Declaration of Helsinki, and ap-proved by the institutional review board of the University of Maia, Maia, Portugal (protocol code: #210/2024; approval date: 28 May 2024).

### 2.2. Protocol

The preseason test session began with a standard warm-up (including mobility exercises for the lower-limb muscles, running technique drills, and two submaximal 10 m sprints) designed for speed-focused days [26], administered by a strength and conditioning coach. The meteorological conditions were sunny with low humidity, and the test was performed on an artificial turf surface. Participants performed two repetitions which could be up to a maximum of four repetitions of the 5-0-5 test, with three minutes of rest between repetitions. For example, if a player slipped or felt that a repetition was not adequate, they were allowed up to two additional attempts. However, only two repetitions were considered valid for analysis, and repetitions not meeting these criteria were automatically excluded and omitted from the dataset. As for the execution of the 5-0-5 test, the player begins the test from a standing start position. At a self-selected moment, the player accelerates maximally along a straight line and runs through the timing gates positioned 10 m from the starting line. After crossing the 10 m timing gates, the player has 5 m available to decelerate and perform a 180° change of direction, making mandatory contact with one foot on the 15 m line marker. Immediately after the turn, the player re-accelerates maximally until passing through the timing gates once again.

In contrast, in the modified 5-0-5 test the timing gates are placed 0.5 m in front of the starting line. Players must accelerate maximally along a linear path, with the turning point located 5 m beyond the timing gates. Consequently, players have substantially less distance to accelerate compared with the 15-0-5 test. Players must decelerate and execute the 180° turning maneuver, making obligatory one-foot contact with the designated turning line, before re-accelerating maximally until they pass through the timing gates for the second time.

### 2.3. Procedures

Locomotor activity was monitored using a portable 10 Hz Global Positioning System (GPS) device (Catapult Vector S7, Catapult Sports, Melbourne, Australia), which has been certified by FIFA (certification number: 1003407) and validated for measuring ACC and DEC [27]. The same GPS was used for the same player throughout the season for reliability issues. The analysis of ACC and DEC was performed using the recurring Catapult Accel Set 2, which utilizes the 10 Hz GPS data to specifically analyze ACC and DEC. The criteria for identifying ACC and DEC efforts using GEN2 are as follows:

- Only data points where velocity exceeds 5 km/h are included. This excludes low-speed movements from the analysis and enhances accuracy.
- Acceleration efforts are calculated using multiple consecutive data points (by default, 9 data points sampled at 0.1-s interval, equivalent to 0.8 s).
- The algorithm fits a linear regression line through the selected data points, and the slope of this line determines the acceleration value.
- To ensure that the effort represents a true, smooth acceleration, any fitted line with a correlation coefficient lower than 0.8 is discarded (excluded from reporting). This filters out noisy or low-quality data.
- A minimum interval of 1 s must separate distinct acceleration efforts. If a new effort satisfies all other criteria but occurs within less than 1 s of a previous effort, the two efforts are merged and reported as a single event. The 1-s minimum interval rule applies only between efforts of the same type (i.e., acceleration-to-acceleration or deceleration-to-deceleration) and does not apply between an acceleration and a deceleration, or vice versa.

**Figure 1:**
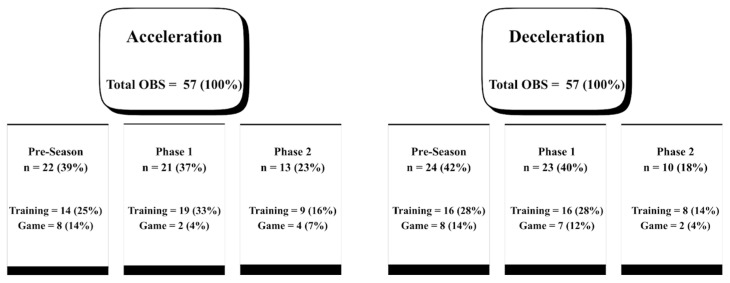
A schematic representation of the occurrences of maximal acceleration and deceleration efforts throughout the 2024/2025 season. Occurrences were stratified according to the competitive phase in which they were observed and, more specifically, by the type of activity (training or match). Total OBS = total number of observations; Pre-Season = The period from 1 July until (but excluding) the date of the first official match of the season (13 August 2024); Phase 1 = the first competitive phase, from the first match of the season until 31 December; Phase 2 = the second competitive phase, from 1 January until the end of the season (30 April 2025).

To avoid spikes caused by GPS error rather than a true performance of the athlete, the average of the three highest values recorded during the season (whether in training sessions or matches) were calculated, excluding any value that was more than 1 m/s^2^ higher than the second-highest value [28]. These values were referred to as maximum acceleration (ACC_max_) or maximum deceleration (DEC_max_). Moreover, Maximum (%) is defined as the ratio between the maximum value observed in the respective condition during the 5-0-5 test (e.g., ACC_S, DEC_COD_ and ACC_COD) and the average of the three highest values recorded throughout the season. The occurrence values of the seasonal maximum values can be visualized in Figure 1.

### 2.4. Statistical Analysis

Raw data were organized and treated in Microsoft Excel (Microsoft Corporation; Version 16.89.1). After that, a statistical analysis was conducted with JASP statistical software (version 0.95.4, University of Amsterdam, Amsterdam, The Netherlands). Before conducting statistical analyses, data normality was assessed both visually (using Q-Q plots and histograms) and objectively using the Shapiro-Wilk test. Paired-sample *t*-test was applied using the average of the 2 repetitions and the best repetition of the 5-0-5 test compared to the maximum throughout the season. Moreover, *p*-values were adjusted for multiple comparisons using the Bonferroni method within each comparison set (adjusted α = 0.025). Adjusted *p*-values were compared against the standard significance level of *p* < 0.05. Correlations were performed using Pearson’s coefficients, for normally distributed data. The correlation coefficients were classified as weak (<0.3), moderate (≥0.3–0.7), and strong (≥0.7) [29]. Cohen’s d effect sizes were calculated for each comparison and categorized as trivial (<0.2), small (≥0.2–0.6), moderate (≥0.6–1.2), large (≥1.2–2.0), very large (≥2.0–4.0) and extremely large (≥4.0) [30]. To assess repeatability, an intraclass correlation coefficient (ICC3.1) analysis was performed between the two repetitions. The ICCs were classified as poor (<0.5), moderate (≥0.5–0.75), good (≥0.75–0.9) and excellent (≥ 0.9) [31]. For each player, the coefficient of variation (CV) was calculated using the 2 repetitions as well as the standard error of measurement (SEM) [32,33].

## 3. Results

### 3.1. Repeatability Analysis

**Figure 2:**
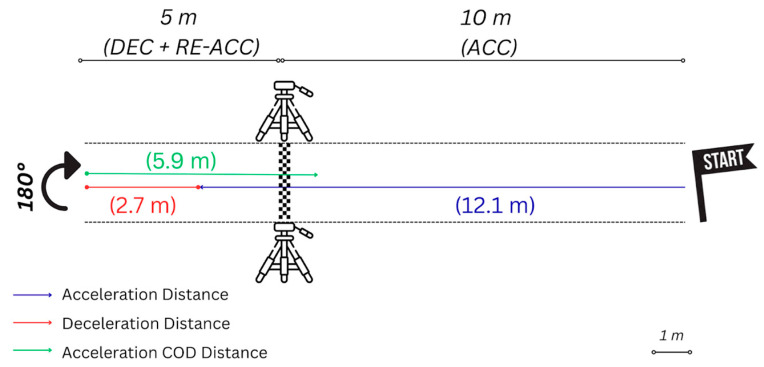
A representation of the setup for the 5-0-5 test. This setup encompasses a 10 m acceleration zone [10 m (ACC)] and a 5 m distance to perform the change of direction maneuver, divided into deceleration and re-acceleration phases [5 m (DEC + RE-ACC)]. On average, players accelerated for 12.1 m (blue arrow), decelerated for 2.7 m (red arrow) and re-accelerated for 5.9 m (green arrow).

Regarding the kinematic characteristics of the 5-0-5 test (Figure 2), the results revealed that, on average, football players required 12.1 m to accelerate, 2.7 m to decelerate, and 5.9 m to re-accelerate. The results of the repeatability analysis between the two repetitions can be seen in Table 1. Briefly, the absolute ACC and DEC parameters showed a relatively low variability (CV: ~5%), and good repeatability for absolute ACC_S (ICC = 0.73).

For the ACC_S condition, most parameters demonstrated good repeatability (Starting speed: ICC = 0.73; Distance: ICC = 0.75; % Maximum: ICC = 0.81), while the remaining variables showed moderate (Absolute: ICC = 0.73) and poor repeatability (Duration: ICC = 0.40). Regarding the DEC_COD parameters, the majority exhibited a poor repeatability magnitude (Absolute: ICC = −0.49; Starting speed: ICC = 0.48; Distance: ICC = −0.45), whereas Duration (ICC = −0.49) and % Maximum (ICC = 0.76) presented moderate and good repeatability, respectively. For the ACC_COD condition, poor repeatability was observed for Absolute (ICC = 0.29) and Starting speed (ICC = −1.09), moderate repeatability for Duration (ICC = 0.64) and Distance (ICC = 0.59), and good repeatability for % Maximum (ICC = 0.85).

### 3.2. Correlation Analysis

The correlations between both the average of the 2 repetitions and the best repetition of the 5-0-5 test are depicted in Table 2 and Table 3, respectively. Significant moderate correlations were only observed between average 5-0-5 ACC_S vs. ACC_max_ (r = 0.48; *p* = 0.040) and best 5-0-5 ACC_S vs. ACC_max_ (r = 0.50; *p* = 0.028).

### 3.3. Comparison of the Average and the Season’s Maximum

Regarding the comparison between the average of the 2-repetitions 5-0-5 test (Table 4) score with the maximum recorded throughout the season, a statistical significant difference was observed with large effect sizes for the comparisons between absolute values of ACC_S vs. ACC_max_ (*p* = 0.004; d = 1.24), as well as between normalized values of ACC_S vs. ACC_max_ (*p* = 0.004; d = 1.31). Moreover, moderate effect sizes were found for the comparisons between absolute values of ACC_COD vs. ACC_max_ (*p* = 0.016; d = 0.76), and between normalized values of ACC_COD vs. ACC_max_ (*p* = 0.024; d = 0.72).

### 3.4. Comparison of the Best Repetition and the Season’s Maximum

Finally, for the comparison between the best repetition obtained during the 5-0-5 test (Table 5) and the maximum recorded throughout the season, a statistically significant difference was observed, accompanied by moderate effect sizes for absolute values between ACC_S vs. ACC_max_ (*p* = 0.004; d = 0.98) and normalized values of ACC_S vs. ACC_max_ (*p* = 0.004; d = 1.05).

## 4. Discussion

To the best of our knowledge, this is the first study to compare the ACC and DEC values obtained during the 5-0-5 test with the highest values achieved by each player throughout the season, as well as to analyze the repeatability of a COD test assessed via GPS devices in football. The results demonstrated (i) that the 5-0-5 COD test can provide repeatable ACC_S values, but not ACC_COD values and that (ii) maximum ACC and DEC values can be obtained during the preseason 5-0-5 test when compared to seasonal ACC_max_ and DEC_max_.

Regarding the repeatability analysis, the parameters of the ACC_S showed in general moderate to good repeatability (expected for duration). Interestingly, the Maximum (%) comparisons across all conditions demonstrated higher repeatability magnitudes than the non-normalized values, whereby maximal performance during the 5-0-5 could be reliably compared to maximum performance throughout the season. This finding supports the use of normalized metrics to facilitate individualized performance analysis, in line with previous research recommendations [34,35]. The observation of poor repeatability magnitudes [ICC: −1.09–0.48] in ACC_COD and DEC_COD indicates possibly no reliability to accurately measure the Staring Speed in the ACC_COD. This result is not unexpected, as players may adopt different movement strategies when performing the 5-0-5 test which will influence the identification of the starting speed of the ACC_COD, since the Catapult algorithm does not overlap ACC and DEC efforts, as it is not physically possible to accelerate and decelerate simultaneously. The algorithm resolves such overlaps by adjusting the start time of the latter effort.

In relation to the correlation between ACC_S and ACC_max_ it was significant but only moderate when using both average 5-0-5 ACC_S (r = 0.48; *p* = 0.040) and best 5-0-5 ACC_S (r = 0.50; *p* = 0.028). This suggests that ACC_max_ performance is likely mediated by additional factors [36,37] not considered within the scope of the present analysis. These findings are partially consistent with those reported in a previous study [18], in which the peak ACC during the pro-agility test (4.67 ± 0.23 m/s^2^) was similar (CI 95%: 0.03; d = 0.09) to the peak ACC recorded during official matches (4.70 ± 0.33 m/s^2^), yet substantially different (CI 95%: 0.31; d = 1.30) when compared with training sessions (4.98 ± 0.22 m/s^2^). However, the authors of that study employed a different COD protocol, which may have influenced the magnitude of the peak ACC values, resulting in lower estimates than the average ACC_S (4.75 ± 0.31 m/s^2^) and best ACC_S (4.88 ± 0.41 m/s^2^) values reported in the present study. The comparison between average 5-0-5 tests captured statistically significant differences for both ACC_S vs. ACC_max_ and ACC_COD vs. ACC_max_, in absolute and normalized metrics. In other words, this study showed that these players display significantly higher magnitudes of ACC during the season compared to the average magnitudes of ACC and re-acceleration observed during the 5-0-5 test. Furthermore, these interactions were found to be of large (ACC_S vs. ACC_max_) and moderate effect size (ACC_COD vs. ACC_max_), which emphasizes the practical relevance of these relationships. The best 5-0-5 Test analysis revealed significance and moderate effect sizes only for ACC_S vs. ACC_max_, indicating that even the best value of ACC attained, from the standing-start position during the 5-0-5 test, is lower compared to the ACC_max_ achieved during the season. Moreover, a previous study has reported superior ACC_max_ during different sprint and acceleration exercises, whereby linear sprints of 30 m or 40 m, or chasing sprints of 30 m resulted in superior ACC_max_ values compared to those obtained in this study for both the 5-0-5 test conditions and the maximum values recorded throughout the season [26]. Therefore, linear sprint efforts may induce higher acceleration magnitudes than the 5-0-5 test; although they could be less specific to the re-acceleration COD in soccer [38,39]. Indeed, in our study the re-acceleration effort immediately following the 180° turn approximates the seasonal maximum (ACC_max_). Given football’s intermittent nature and frequent COD demands [2,38,39], players could exhibit higher ACC capacity post-180° turn compared to linear efforts, as a specific adaptation to the imposed demands. In fact, a previous study reported a greater magnitude of ACC registered during the first phase (linear) of the 5-0-5 test compared to the re-acceleration of participants with “experience” in multiple team sports [40]. Therefore, a greater capacity to re-accelerate compared to the potential to ACC from a standing-start position could be a specific quality of football players. Nonetheless, small magnitude effect sizes were observed for the ACC_COD vs. ACC_max_, therefore suggesting a low practical significance of such interactions. This warrants further investigation as it would be useful to comprehend whether the capacity to ACC could be significantly associated with a preceding DEC and to which extent the 5-0-5 could accurately identify the real ACC_max_ of a soccer player. This could constitute essential information for individualized monitoring and prescription ACC related training loads.

Regarding the DEC metrics, no significant differences were observed between both 5-0-5 repetitions analysis (average and best repetition) and seasonal maximum. However, higher values were observed during the 505 test. The moderate effect size suggests practical relevance, particularly since small differences in COD performance can determine decisive moments in match play. Interestingly, DEC_COD performance was noted to be superior to the DEC_max_, suggesting that the 5-0-5 test performance provides superior DEC intensity stimuli compared to what players might experience throughout the season. This has important implications for training prescription, especially for coaches who may use game-reference values to normalize training intensity [41], therefore underestimating the players’ real maximum capacity. Furthermore, the linear DEC_max_ has been shown to be a critical component of COD performance [42], therefore it should be adequately addressed in order to improve COD capacity. In practical terms, coaches could use the best ACC_COD and DEC_COD values obtained early in the pre-season as a reference for monitoring and individualized training prescription, particularly for newly integrated players, with no retrospective performance data available to benchmark the players’ maximum performance. The advantages of analyzing ACC and DEC efforts through a normalized approach are emphasized when comparing to the absolute fixed thresholds usually applied to define high-intensity DEC (<−3 m/s^2^) and ACC efforts (>3 m/s^2^). Indeed, our data demonstrated that, even when using the average comparison, players achieved approximately 63–68% of the ACC and 123% of the DEC values, when compared to the absolute thresholds of >3 m/s^2^ and <–3 m/s^2^, respectively.

## 5. Study Limitations

This study has some limitations. Firstly, ACC is a vector quantity, it encompasses both magnitude and directional components. However, since GPS devices typically treat ACC as a scalar variable, they fail to account for COD, thereby compromising the accurate assessment of ACC during COD movements, particularly in sharper maneuvers (e.g., a 180° cut) [17]. As a result, successive derivations amplify measurement noise and reduce the signal-to-noise ratio of ACC data. Moreover, extensive research on GPS-based tracking systems has demonstrated that measurement reliability decreases as a function of movement intensity, whereas validity tends to increase with greater distances covered [43]. Furthermore, measurement accuracy has been reported to be higher during linear locomotion compared with multidirectional movements [14,44,45]. These limitations compromise the accurate assessment of short, high-intensity efforts such as rapid multidirectional DEC and ACC. Such inaccuracies are likely attributable to the computational process by which ACC values are derived from speed data [17]. Despite these methodological constraints, GPS devices remain widely employed in football contexts due to their ease of implementation and relatively low cost compared with more sophisticated motion analysis systems.

Secondly, it must be taken into consideration that the ICC values could be overestimated since they represent an intra-session repeatability format. Subsequently, the two repetitions of the 5-0-5 test were conducted under, practically, the same conditions providing more stable results between trials compared to a test-retest reliability assessments performed on different days. It is important to keep in mind the lack of inter-session reliability analysis and the potential influence of environmental factors (e.g., weather, surface) when trying to replicate this study. The negative ICC values suggest that variability within the same athlete was greater than variability between athletes, meaning that an athlete could adopt different movement strategies across repetitions, resulting in noticeable differences in DEC_COD distances. In other words, athletes tend to differ less from each other than from their own performance across repetitions. Even for variables with relatively high CV values, the negative ICCs indicate that this within-athlete variability remained higher than the differences observed between athletes for the parameter in question.

Finally, a relatively small sample size may be considered a limitation, potentially reducing the statistical power of the analyses. However, the post hoc power analysis indicated that, on average, statistical power for the two comparisons was adequate (>70%). Nevertheless, the results should not be generalized to other populations, such as female players or players from different competitive levels. Nevertheless, it is operationally challenging to access football environments for research purposes, particularly when involving high-level teams. Despite this limitation, the current study offers a reproducible methodological framework that can be replicated in other football populations and subsequently integrated into systematic reviews or meta-analyses to foster a more accurate and comprehensive interpretation of these issues.

## 6. Conclusions

This study provides relevant and meaningful insights into change-of-direction performance in football players, particularly within the context of the 5-0-5 test. Firstly, our findings suggest that ACC and DEC capacities exhibited during the best repetition of 5-0-5 test at the onset of the pre-season may be comparable to the respective maximum values recorded throughout the season. Moreover, the study demonstrates good repeatability for the normalized variables (expressed as a percentage of the maximum values obtained during the season). Therefore, coaches could use these variables for benchmarking and a tailored approach to external load monitoring and training prescription. The present study demonstrated that football players exhibited a higher value of ACC following the DEC and turning phase than the ACC magnitude achieved during the initial linear ACC phase of the test.

## Figures and Tables

**Table 1:** Within-session intraclass correlation coefficients (ICCs) with 95% confidence intervals (95% CI) for the measured variables in the 5-0-5 change of direction (COD) test.

		Average ± SD				
		Rep 1 (*n* = 19)	Rep 2 (*n* = 19)	ICC [95% Confidence Interval]	CV (%)	SEM
ACC_S	Absolute (m/s^2^)	4.80 ± 0.32	4.75 ± 0.54	0.73 [0.26–0.90]	4.6	0.1
	Duration (s)	2.06 ± 0.10	2.16 ± 0.23	0.40 [−0.39–0.76]	3.1	0.1
	Starting Speed (m/s)	0.69 ± 0.24	0.62 ± 0.11	0.75 [0.36–0.91]	17.9	0.0
	Distance (m)	12.06 ± 0.48	12.18 ± 0.39	0.78 [0.43–0.92]	1.8	0.1
	Maximum (%)	89.87 ± 7.27	88.83 ± 10.93	0.81 [0.48–0.93]	4.5	1.7
DEC_COD	Absolute (m/s^2^)	−6.58 ± 0.32	− 6.41 ± 0.44	−0.49 [−2.90–0.44]	−5.2	0.4
	Duration (s)	1.07 ± 0.23	1.07 ± 0.15	−0.54 [−3.86–0.45]	12.7	0.2
	Starting Speed (m/s)	6.55 ± 0.18	6.59 ± 0.16	0.48 [0.40–0.81]	1.5	0.1
	Distance (m)	2.60 ± 0.64	2.80 ± 0.58	−0.45 [−3.06–0.47]	20.6	0.7
	Maximum (%)	104.52 ± 12.51	101.65 ± 11.58	0.76 [0.38–0.91]	5.2	2.7
ACC_COD	Absolute (m/s^2^)	4.81 ± 0.30	4.99 ± 0.41	0.29 [−0.69–0.72]	5.3	0.2
	Duration (s)	1.21 ± 0.15	1.19 ± 0.17	0.64 [0.05–0.86]	7.9	0.1
	Starting Speed (m/s)	1.20 ± 0.53	1.44 ± 0.73	−1.09 [−4.99–0.24]	41.9	0.9
	Distance (m)	5.90 ± 0.73	5.82 ± 0.94	0.59 [−0.13–0.85]	8.8	0.3
	Maximum (%)	90.57 ± 12.34	93.82 ± 12.75	0.85 [0.60–0.94]	5.3	1.9

Legend: ACC_S = Highest acceleration value achieved when initiating the effort from a standing start position (i.e., at the beginning of the test) until the player begins the deceleration phase; DEC_COD = The highest deceleration value recorded between the onset of deceleration following the initial standing-start acceleration and the subsequent re-acceleration required to execute the 180° turn; ACC_COD = The highest acceleration value achieved immediately following a deceleration phase. In the context of the test, this corresponds to the re-acceleration action performed immediately after the 180° turn at the 15 m mark. Absolute (m/s^2^) = maximum value recorded for the respective condition; Duration (s) = total time of the respective effort; Starting Speed (m/s) = speed immediately preceding the onset of the respective effort; Distance (m) = distance covered during the execution of the respective effort; % Maximum = ratio between the maximum value observed in the respective condition and the average of the three highest values recorded throughout the season; CV = coefficient of variation; SEM = standard error of measurement.

**Table 2:** The correlations (Pearson r) between the average of the two repetitions of the 5-0-5 test and the maximum acceleration and deceleration values recorded throughout the season.

	ACC_max_		DEC_max_	
	Pearson (*r*)	*p*-Value	Pearson (*r*)	*p*-Value
ACC_S	0.48 *	0.040		
ACC_COD	−0.04	0.859		
DEC_COD			0.04	0.881

Legend: ACC_S = The highest acceleration value achieved when initiating the effort from a standing start position (i.e., at the beginning of the test) until the player begins the deceleration phase; DEC_COD = The highest deceleration value recorded between the onset of deceleration following the initial standing-start acceleration and the subsequent re-acceleration required to execute the 180° turn; ACC_COD = The highest acceleration value achieved immediately following a deceleration phase. In the context of the test, this corresponds to the re-acceleration action performed immediately after the 180° turn at the 15 m mark. Pearson correlations are expressed as r. * *p* < 0.05 = Significant correlations were considered at *p* < 0.05.

**Table 3:** Correlations (Pearson r) between the best repetition of the 5-0-5 test and the maximum acceleration and deceleration values recorded throughout the season.

	ACC_max_		DEC_max_	
	Pearson (*r*)	*p*-Value	Pearson (*r*)	*p*-Value
ACC_S	0.50 *	0.028		
ACC_COD	0.01	0.980		
DEC_COD			0.24	0.317

Legend: ACC_S = The highest acceleration value achieved when initiating the effort from a standing start position (i.e., at the beginning of the test) until the player begins the deceleration phase; DEC_COD = The highest deceleration value recorded between the onset of deceleration following the initial standing-start acceleration and the subsequent re-acceleration required to execute the 180° turn; ACC_COD = The highest acceleration value achieved immediately following a deceleration phase. In the context of the test, this corresponds to the re-acceleration action performed immediately after the 180° turn at the 15 m mark. Pearson correlations are expressed as r. * *p* < 0.05 = Significant correlations were considered at *p* < 0.05.

**Table 4:** A comparison between the average of the two repetitions of the 5-0-5 test and the maximum acceleration and deceleration values recorded throughout the season.

	Average 5-0-5 Test vs. Maximum Values	Average ± SD	t	*p*	Effect Size [95% Confidence Interval]
ACC_S vs. ACC_max_ (*n* = 19)	ACC_S (m/s^2^)	4.75 ± 0.40	−5.41	0.004	1.24 [0.63–1.83]
	ACC_max_ (m/s^2^)	5.36 ± 0.54			
	ACC_S (%)	89.04 ± 8.35	−5.72	0.004	1.31 [0.68–1.92]
	ACC_max_ (%)	100.00 ± 0.00			
DEC_COD vs. DEC_max_ (*n* = 19)	DEC_COD (m/s^2^)	−6.47 ± 0.26	−0.82	1.0	0.19 [0.27–0.64]
	DEC_max_ (m/s^2^)	−6.35 ± 0.61			
	DEC_COD (%)	102.80 ± 10.62	1.15	1.0	0.26 [0.72–0.20]
	DEC_max_ (%)	100.00 ± 0.00			
ACC_COD vs. ACC_max_ (*n* = 19)	ACC_COD (m/s^2^)	4.78 ± 0.54	−3.33	0.016	0.76 [0.24–1.27]
	ACC_max_ (m/s^2^)	5.36 ± 0.54			
	ACC_COD (%)	91.67 ± 11.63	−3.12	0.024	0.72 [0.20–1.22]
	ACC_max_ (%)	100.00 ± 0.00			
ACC_S vs. ACC_COD (*n* = 19)	ACC_S (m/s^2^)	4.75 ± 0.40	−0.30	1.0	0.07 [0.38–0.52]
	ACC_COD (m/s^2^)	4.78 ± 0.54			
	ACC_S (%)	89.04 ± 8.35	−1.45	1.0	0.33 [0.14–0.79]
	ACC_COD (%)	91.67 ± 11.63			

Legend: ACC_S = The highest acceleration value achieved when initiating the effort from a standing start position (i.e., at the beginning of the test) until the player begins the deceleration phase; ACC_max_ = The average of the three highest acceleration values recorded throughout the season; DEC_COD = The highest deceleration value recorded between the onset of deceleration following the initial standing-start acceleration and the subsequent re-acceleration required to execute the 180° turn; DEC_max_ = The average of the three highest deceleration values recorded throughout the season ACC_COD = The highest acceleration value achieved immediately following a deceleration phase. In the context of the test, this corresponds to the re-acceleration action performed immediately after the 180° turn at the 15 m mark.

**Table 5:** A comparison between the best repetition of the 5-0-5 test and the maximum acceleration and deceleration values recorded throughout the season.

	Best 5-0-5 Test vs. Maximum Values	Average ± SD	t	*p*	Effect Size [95% Confidence Interval]
ACC_S vs. ACC_max_ (*n* = 19)	ACC_S (m/s^2^)	4.88 ± 0.31	−4.28	0.004	0.98 [0.42–1.52]
	ACC_max_ (m/s^2^)	5.36 ± 0.54			
	ACC_S (%)	91.94 ± 7.65	−4.59	0.004	1.05 [0.48–1.61]
	ACC_max_ (%)	100.00 ± 0.00			
DEC_COD vs. DEC_max_ (*n* = 19)	DEC_COD (m/s^2^)	−6.69 ± 0.36	−1.94	0.272	0.45 [0.03–0.91]
	DEC_max_ (m/s^2^)	−6.35 ± 0.61			
	DEC_COD (%)	106.52 ± 13.24	2.15	0.184	0.49 [0.96–0.01]
	DEC_max_ (%)	100.00 ± 0.00			
ACC_COD vs. ACC_max_ (*n* = 19)	ACC_COD (m/s^2^)	5.04 ± 0.39	−2.12	0.208	0.49 [0.00–0.96]
	ACC_max_ (m/s^2^)	5.36 ± 0.54			
	ACC_COD (%)	94.94 ± 12.56	−1.76	0.384	0.40 [0.07–0.87]
	ACC_max_ (%)	100.00 ± 0.00			
ACC_S vs. ACC_COD (*n* = 19)	ACC_S (m/s^2^)	4.88 ± 0.41	−1.65	0.464	0.38 [0.09–0.84]
	ACC_COD (m/s^2^)	4.78 ± 0.54			
	ACC_S (%)	91.94 ± 7.65	−1.56	0.548	0.36 [0.11–0.82]
	ACC_COD (%)	94.94 ± 12.56			

Legend: ACC_S = The highest acceleration value achieved when initiating the effort from a standing start position (i.e., at the beginning of the test) until the player begins the deceleration phase; ACC_max_ = The average of the three highest acceleration values recorded throughout the season; DEC_COD = The highest deceleration value recorded between the onset of deceleration following the initial standing-start acceleration and the subsequent re-acceleration required to execute the 180° turn; DEC_max_ = The average of the three highest deceleration values recorded throughout the season; ACC_COD = The highest acceleration value achieved immediately following a deceleration phase. In the context of the test, this corresponds to the re-acceleration action performed immediately after the 180° turn at the 15 m mark.

## Data Availability

Data are not publicly available due to privacy and ethical restrictions.

## Abbreviations

The following abbreviations are used in this manuscript:

ACC	Acceleration
ACC_COD	The highest acceleration value achieved immediately following a deceleration phase. In the context of the test, this corresponds to the re-acceleration action performed immediately after the 180° turn at the 15 m mark.
ACC_max_	The average of the three highest acceleration values recorded during the season (whether in training sessions or matches)
ACC_S	Highest acceleration value achieved when initiating the effort from a standing start position (i.e., at the beginning of the test) until the player begins the deceleration phase.
COD	Change of direction
DEC_COD	The highest deceleration value recorded between the onset of deceleration following the initial standing-start acceleration and the subsequent re-acceleration required to execute the 180° turn.
DEC_max_	The average of the three highest deceleration values recorded during the season (whether in training sessions or matches).
GPS	Global Positioning System
ICC	Intraclass Correlation Coefficient
95% CI	95% Confidence Interval
Maximum(%)	Ratio between the maximum value observed in the respective condition and the average of the three highest values recorded throughout the season

## References

[1] Chaouachi A., Manzi V., Chaalali A., Wong D.P., Chamari K., Castagna C. (2012). Determinants Analysis of Change-of-Direction Ability in Elite Soccer Players. J. Strength Cond. Res..

[2] Loturco I., Jeffreys I., Abad C.C.C., Kobal R., Zanetti V., Pereira L.A., Nimphius S. (2020). Change-of-Direction, Speed and Jump Performance in Soccer Players: A Comparison across Different Age-Categories. J. Sports Sci..

[3] Taylor J.M., Cunningham L., Hood P., Thorne B., Irvin G., Weston M. (2019). The Reliability of a Modified 505 Test and Change-of-Direction Deficit Time in Elite Youth Football Players. Sci. Med. Footb..

[4] Negra Y., Chaabene H., Amara S., Jaric S., Hammami M., Hachana Y. (2017). Evaluation of the Illinois Change of Direction Test in Youth Elite Soccer Players of Different Age. J. Hum. Kinet..

[5] Quintero-Illera J.L., Nevado F., Zarzuela-Martín R., López-Del Campo R., Cuadrado-Peñafiel V. (2025). Impact of Acceleration and Acceleration-Initial Speed Profiles on Team Success in LaLiga. Appl. Sci..

[6] Oliva-Lozano J.M., Martínez-Puertas H., Fortes V., Campo R.L.-D., Resta R., Muyor J.M. (2023). Is There Any Relationship between Match Running, Technical-Tactical Performance, and Team Success in Professional Soccer? A Longitudinal Study in the First and Second Divisions of LaLiga. Biol. Sport.

[7] Nimphius S., Callaghan S.J., Bezodis N.E., Lockie R.G. (2018). Change of Direction and Agility Tests: Challenging Our Current Measures of Performance. Strength Cond. J..

[8] Ryan C., Uthoff A., McKenzie C., Cronin J. (2022). Traditional and Modified 5-0-5 Change of Direction Test: Normative and Reliability Analysis. Strength Cond. J..

[9] Haugen T.A., Tønnessen E., Svendsen I.S., Seiler S. (2014). Sprint Time Differences between Single- and Dual-Beam Timing Systems. J. Strength Cond. Res..

[10] Sheppard J.M., Young W.B. (2006). Agility Literature Review: Classifications, Training and Testing. J. Sports Sci..

[11] Varley M.C., Fairweather I.H., Aughey R.J. (2012). Validity and Reliability of GPS for Measuring Instantaneous Velocity during Acceleration, Deceleration, and Constant Motion. J. Sports Sci..

[12] Johnston R.J., Watsford M.L., Kelly S.J., Pine M.J., Spurrs R.W. (2014). Validity and Interunit Reliability of 10 Hz and 15 Hz GPS Units for Assessing Athlete Movement Demands. J. Strength Cond. Res..

[13] Brosnan R.J., Watson G., Stuart W., Twentyman C., Kitic C.M., Schmidt M. (2022). The Validity, Reliability, and Agreement of Global Positioning System Units-Can We Compare Research and Applied Data?. J. Strength Cond. Res..

[14] Vickery W.M., Dascombe B.J., Baker J.D., Higham D.G., Spratford W.A., Duffield R. (2014). Accuracy and Reliability of GPS Devices for Measurement of Sports-Specific Movement Patterns Related to Cricket, Tennis, and Field-Based Team Sports. J. Strength Cond. Res..

[15] Hodder R.W., Ball K.A., Serpiello F.R. (2020). Criterion Validity of Catapult ClearSky T6 Local Positioning System for Measuring Inter-Unit Distance. Sensors.

[16] Clemente F.M., Akyildiz Z., Garrett J., Beato M., Yildiz M., Birlik S., Moran J. (2023). Testing the Peak Running Speed in Analytical and Contextual-Based Scenarios: Applied Research in Young Adult Soccer Players. J. Sports Sci..

[17] Ellens S., Moran C., Varley M.C. (2025). Concurrent Validity and between-Device Reliability of the Catapult Vector S8 GNSS Device. medRxiv.

[18] Silva H., Nakamura F.Y., Roriz P., Marcelino R. (2025). Are the Peak Accelerations and Decelerations Registered during the pro-Agility Test Replicated during Matches and Training Sessions?. Int. J. Perform. Anal. Sport..

[19] Çınarlı F.S., Şahin Kafkas A., Kafkas M.E. (2018). Relationship between Linear Running and Change of Direction Performances of Male Soccer Players. Turk. J. Sport Exerc..

[20] Koral J., Lloria Varella J., Lazaro Romero F., Foschia C. (2021). Effects of Three Preseason Training Programs on Speed, Change-of-Direction, and Endurance in Recreationally Trained Soccer Players. Front. Physiol..

[21] Michaelides M.A., Parpa K.M., Zacharia A.I. (2021). Effects of an 8-Week Pre-Seasonal Training on the Aerobic Fitness of Professional Soccer Players. J. Strength Cond. Res..

[22] Caldwell B.P., Peters D.M. (2009). Seasonal Variation in Physiological Fitness of a Semiprofessional Soccer Team. J. Strength Cond. Res..

[23] Springham M., Williams S., Waldron M., Burgess D., Newton R.U. (2020). Large Reductions in Match Play Physical Performance Variables Across a Professional Football Season with Control for Situational and Contextual Variables. Front. Sports Act. Living.

[24] McKay A.K.A., Stellingwerff T., Smith E.S., Martin D.T., Mujika I., Goosey-Tolfrey V.L., Sheppard J., Burke L.M. (2022). Defining Training and Performance Caliber: A Participant Classification Framework. Int. J. Sports Physiol. Perform..

[25] White A., Hills S.P., Cooke C.B., Batten T., Kilduff L.P., Cook C.J., Roberts C., Russell M. (2018). Match-Play and Performance Test Responses of Soccer Goalkeepers: A Review of Current Literature. Sports Med..

[26] Pimenta R., Maia F., Silva H., Nakamura F.Y. (2025). The Speed Dynamics of Different Sprint and Acceleration Exercises Applied during Football Training. Sci. Rep..

[27] Crang Z.L., Duthie G., Cole M.H., Weakley J., Hewitt A., Johnston R.D. (2024). The Validity of Raw Custom-Processed Global Navigation Satellite Systems Data during Straight-Line Sprinting across Multiple Days. J. Sci. Med. Sport..

[28] Silva H., Nakamura F.Y., Ribeiro J., Asian-Clemente J., Roriz P., Marcelino R. (2023). Using Minimum Effort Duration Can Compromise the Analysis of Acceleration and Deceleration Demands in Football. Int. J. Perform. Anal. Sport.

[29] Sheskin D.J. (2020). Handbook of Parametric and Nonparametric Statistical Procedures.

[30] Hopkins W.G., Marshall S.W., Batterham A.M., Hanin J. (2009). Progressive Statistics for Studies in Sports Medicine and Exercise Science. Med. Sci. Sports Exerc..

[31] Koo T.K., Li M.Y. (2016). A Guideline of Selecting and Reporting Intraclass Correlation Coefficients for Reliability Research. J. Chiropr. Med..

[32] Atkinson G., Nevill A.M. (1998). Statistical Methods for Assessing Measurement Error (reliability) in Variables Relevant to Sports Medicine. Sports Med..

[33] Hopkins W.G. (2000). Measures of Reliability in Sports Medicine and Science. Sports Med..

[34] Pimenta R., Antunes H., Ribeiro J., Nakamura F.Y. (2025). Should GPS Data Be Normalized for Performance and Fatigue Monitoring in Soccer? A Theoretical-Practical Discussion on High-Speed Running. Front. Sports Act. Living.

[35] Pimenta R., Antunes H., Ribeiro J., Yuzo Nakamura F. (2025). Should GPS Data Be Normalized for Performance and Fatigue Monitoring in Soccer? A Theoretical–practical Discussion on Sprinting. Ger. J. Exerc. Sport. Res..

[36] Murphy A.J., Lockie R.G., Coutts A.J. (2003). Kinematic Determinants of Early Acceleration in Field Sport Athletes. J. Sports Sci. Med..

[37] Buchheit M., Samozino P., Glynn J.A., Michael B.S., Al Haddad H., Mendez-Villanueva A., Morin J.B. (2014). Mechanical Determinants of Acceleration and Maximal Sprinting Speed in Highly Trained Young Soccer Players. J. Sports Sci..

[38] Dos’Santos T., Cowling I., Challoner M., Barry T., Caldbeck P. (2022). What Are the Significant Turning Demands of Match Play of an English Premier League Soccer Team?. J. Sports Sci..

[39] Deutsch J.-P., Donath L., Braunstein B., Rein R. (2025). Frequency and Intensity of Change of Directions in German Bundesliga Soccer. Sci. Med. Footb..

[40] Buchheit M., Eriksrud O. (2024). Maximal Locomotor Function in Elite Football: Protocols and Metrics for Acceleration, Speed, Deceleration, and Change of Direction Using a Motorized Resistance Device. Sport Perform. Sci. Rep..

[41] Ravé G., Granacher U., Boullosa D., Hackney A.C., Zouhal H. (2020). How to Use Global Positioning Systems (GPS) Data to Monitor Training Load in the “Real World” of Elite Soccer. Front. Physiol..

[42] Harper D.J., Jordan A.R., Kiely J. (2021). Relationships Between Eccentric and Concentric Knee Strength Capacities and Maximal Linear Deceleration Ability in Male Academy Soccer Players. J. Strength Cond. Res..

[43] Jennings D., Cormack S., Coutts A.J., Boyd L., Aughey R.J. (2010). The Validity and Reliability of GPS Units for Measuring Distance in Team Sport Specific Running Patterns. Int. J. Sports Physiol. Perform..

[44] Buchheit M., Allen A., Poon T.K., Modonutti M., Gregson W., Di Salvo V. (2014). Integrating Different Tracking Systems in Football: Multiple Camera Semi-Automatic System, Local Position Measurement and GPS Technologies. J. Sports Sci..

[45] Ogris G., Leser R., Horsak B., Kornfeind P., Heller M., Baca A. (2012). Accuracy of the LPM Tracking System Considering Dynamic Position Changes. J. Sports Sci..

